# Implication of genetic variants near *SLC30A8, HHEX, CDKAL1, CDKN2A/B, IGF2BP2, FTO, TCF2*, *KCNQ1*, and *WFS1 *in Type 2 Diabetes in a Chinese population

**DOI:** 10.1186/1471-2350-11-81

**Published:** 2010-05-28

**Authors:** Xueyao Han, Yingying Luo, Qian Ren, Xiuying Zhang, Fang Wang, Xiuqin Sun, Xianghai Zhou, Linong Ji

**Affiliations:** 1 Department of Endocrinology and Metabolism, Peking University People's Hospital, Peking University Diabetes Center, No 11, Xizhimen South Street, Beijing, China, 100044

## Abstract

**Background:**

Recently, several genome-wide and candidate gene association studies have identified many novel genetic loci for type 2 diabetes (T2D); among these genes, *CDKAL1, IGF2BP2, SLC30A8, CDKN2A/B, HHEX, FTO, TCF2, KCNQ1, and WFS1 *are the most important. We aimed to determine the effects of these genetic loci associated with T2D in the Chinese Han population of China.

**Methods:**

Single-nucleotide polymorphisms (SNPs) in or near *CDKAL1, IGF2BP2, SLC30A8, CDKN2A/B, HHEX, FTO, TCF2, KCNQ1*, and *WFS1 *genes were genotyped in a case-control Chinese Han sample living in Beijing, China involving 1024 patients with T2D and 1005 control subjects.

**Results:**

In Chinese Han, we replicated the associations between 7 genetic loci and T2D, with risk allele-specific odds ratios (ORs) as follows: 1.27 (95% CI, 1.11-1.45; p = 0.0008) for *CDKAL1-*rs10946398, 1.26 (95% CI, 1.08-1.47; p = 0.003) for *IGF2BP2-*rs4402960, 1.19 (95% CI, 1.04-1.37; p = 0.009) for *SLC30A8-*rs13266634, 1.22 (95% CI, 1.06-1.41; p = 0.005) for *CDKN2A/B-*rs10811661, 1.20 (95% CI, 1.01-1.42; p = 0.03) for *HHEX-*rs5015480, 1.37 (95% CI, 1.19-1.69; p = 1.0 × 10^-4^) for *KCNQ1*-rs2237892, and 1.24 (95% CI, 1.01-1.52; p = 0.046) for *FTO-*rs8050136 after adjustment for age, gender, and body mass index. Not only did an association between *WFS1*-rs6446482 and early-onset T2D exist in the subgroup analysis, but *TCF2-*rs7501939 and *WFS1*-rs6446482 were also confirmed to confer risk for T2D in this meta-analysis. Moreover, the relationship between *FTO-*rs8050136 and body mass index, together with the effect of *CDKAL1-*rs10946398 on beta cell function, was also observed in the control individuals.

**Conclusions:**

Our findings support the important contribution of these genetic loci to susceptibility for T2D in the Chinese Han population in Beijing of China.

## Background

Genome-wide association studies (GWAS) have identified many novel susceptibility genes for type 2 diabetes (T2D) since 2007. In the early GWAS, common variants in *IGF2BP2*, *CDKAL1*, *SLC30A8*, *HHEX*, *CDKN2A/B*, *FTO*, and *TCF2 *loci were reported to increase the risk of T2D in Caucasians [1-6], and *KCNQ1 *was recently discovered as a new diabetogenic gene in Japanese samples [7]. In addition, the Wolfram syndrome 1(*WFS1*) gene is one of the novel susceptibility genes for T2D identified by the candidate gene approach in Caucasians until recently [8]. Compared with the susceptibility genes for T2D revealed in the following meta-analysis of GWAS [9], these 9 genes, which were discovered in relatively smaller samples, might represent the most diabetogenic genes and have larger effect sizes in the studied populations. Although these associations have been replicated in Caucasians, the roles of some loci remain less clear in Chinese. For example, in the Chinese Han population, the associations between single nucleotide polymorphisms (SNPs) at the *HHEX, IGF2BP2, TCF2, and FTO *loci with T2D were not consistently replicated [6,10-15], and the findings in previous studies involving Caucasians for SNPs at the *WFS1 *locus have not been confirmed [12]. Moreover, in contrast to the large-scale association studies in a European Caucasian population, such studies in China often have a relatively small sample size, and meta-analysis is becoming particularly useful to evaluate the effects of these genes.

Therefore, we hypothesized that the aforementioned genes were also the most important susceptibility genes for T2D in the Chinese Han population living in China. We aimed to test this hypothesis in a case-control study and perform a meta-analysis of all published data for these genetic loci in the Chinese Han population to fully evaluate their effects.

## Methods

### Subjects

A total of 2029 individuals, including 1024 patients with T2D and 1005 control subjects, were included in the present study. All participants were unrelated and of Northern Han Chinese ancestry residing in the Beijing metropolitan area. The patients were recruited from the Endocrinology and Metabolism Outpatient Clinics of Peking University People's Hospital in Beijing, China, and were diagnosed with T2D in accordance with the 1999 WHO criteria (fasting plasma glucose ≥7.0 mmol/l and/or 2-h plasma glucose ≥11.1 mmol/l) [16]. Patients diagnosed with T2D before 30 years of age, those with an elevated body mass index (BMI; > 35 kg/m^2^), or those with other clinical and genetic features for specific-type diabetes (e.g., maturity onset diabetes of the young [MODY]) were excluded from the study [17,18]. The control subjects were selected from communities near Peking University People's Hospital. The inclusion criteria for the control subjects in this study were as follows: (1) normal glucose regulation confirmed by a 75 g oral glucose tolerance test (OGTT) according to the 1999 WHO criteria (fasting plasma glucose <6.1 mmol/l and 2-h plasma glucose <7.8 mmol/l), (2) no family history of T2D, (3) >40 years of age, and (4) a BMI ≤ 35 kg/m^2^. The clinical features of the participants are summarized in Table 1. Informed consent was obtained from every participant, and the study protocol was approved by the Ethics Committee of Peking University People's Hospital.

**Table 1:** Clinical characteristics of the participants.

Characteristics	Controls	Patients	*P*
Male/Female	343/662	540/484	<0.00001
Age at examination(years)	58 ± 9	56 ± 12	<0.00001
BMI(kg/m^2^)	25.0 ± 3.3	25.0 ± 3.1	0.87
Age of diagnosis(years)	--	49 ± 11	--
Fasting glucose (mmol/l)	5.2 ± 0.4	7.8 ± 2.8	<0.00001
Glucose at 2 h (mmol/l)	5.7 ± 1.2	--	--
Fasting insulin (pmol/l)	53.1(38.0-77.5)	--	--
HOMA-IR	1.60(1.13-2.36)	--	--
HOMA-ß	80.51(57.63-111.72)	--	--

Data are the mean ± SD or median(interquartile range). *p *represents the significance of the differences between the patients and controls.

The gender distribution between the patients and controls was analyzed using Pearson's χ^2 ^test.

Comparison of the quantitative variables between patients and controls was performed using Student's t-test.

### Anthropometric and biological measurements

All of the patients with T2D and control subjects were examined in the morning after an overnight fast for at least 8 hours, with measurements of height, weight, and blood pressure. Blood samples were collected for biochemical measurements of fasting plasma glucose for all the participants, and 2-hour plasma glucose during a 75 g OGTT for the controls. The plasma glucose concentrations were measured by the glucose oxidase-peroxidase method. The fasting serum insulin concentrations were measured by an electrochemiluminescence immunoassay on a Roche Elecsys 2010 Chemistry Analyzer. The homeostasis model assessment of the insulin resistance index (HOMA-IR) and beta-cell function (HOMA-ί) were assessed, as described previously [19].

### Genotyping

Nine SNPs representing nine genes identified in recent GWAS or large candidate gene studies [1-8] were selected, including *SLC30A8-*rs13266634, *HHEX- *rs5015480, *CDKAL1-*rs10946398, *CDKN2A/B-*rs10811661, *IGF2BP2-*rs4402960, *FTO*-rs8050136, *TCF2-*rs7501939, *KCNQ1*-rs2237892, and *WFS1- *rs6446482. Genotyping of these SNPs was performed with an ABI SNaPshot^® ^Multiplex System (Applied Biosystems, Foster City, CA, USA) in the Chinese National Human Genome Center in Shanghai. Genotyping accuracy, as determined from the genotype concordance between duplicate samples (130 samples), was 100%. The genotyping success rate was >95% for the patient and control groups.

### Statistical analysis

Data are given as the means ± SD for quantitative variables with a normal distribution, and as medians (interquartile range) for non-normally distributed data. Fasting serum insulin, HOMA-IR, and HOMA-ί were natural logarithm-transformed to normal distributions before statistical analysis.

Characteristics were compared and tested for significant differences between patients and controls using Student's t test for continuous variables, and using χ^2 ^test for categorical variable. Chi-squared tests were used to determine whether or not individual polymorphisms were in Hardy-Weinberg equilibrium (p > 0.05). The differences in allele and genotype frequencies between the patients with T2D and controls were analyzed using Pearson's χ^2 ^test. Logistic regression analysis was performed to calculate risk allele-specific odds ratios (ORs), 95% confidential intervals (CIs), and corresponding p -values after adjustment for gender, age, and BMI as covariates; under an additive model, 10,000 permutations were used to obtain a true estimate for statistical significance.

Linear regression was applied to test quantitative variables for differences between the genotype groups. SNPs that showed nominal evidence for an association between the age of diagnosis (AOD) for T2D, fasting plasma glucose, fasting serum insulin, HOMA-ί, and HOMA-IR were adjusted by age, gender and BMI. Additionally, BMI was adjusted for age and gender. All analyses were adjusted using linear regression on *a priori *genotypic models (dominant, additive. and recessive). Due to a lack of validity of the large sample χ2 test statistic, only the dominant model was considered for SNPs with ≤10 individuals homozygous for the minor allele (for *WFS1 *and *FTO *loci in the present study).

All statistical tests were performed by PLINK, version 1.06 http://pngu.mgh.harvard.edu/~purcell/plink or SPSS, version 11.5 for Windows (SPSS, Inc., Chicago, IL, USA). A p-value < 0.05 was considered statistically significant (two-tailed).

Power calculations were performed using Quanto software (available at http://hydra.usc.edu/gxe/), and the powers shown in Additional file 1, Table S1 were calculated using ORs from published studies, sample sizes and minor allele frequencies (MAF) in the present study, the prevalence of T2D in Chinese of mainland China [20], and the type 1 error rate (0.05).

Pairwise linkage disequilibrium (LD) coefficients (r^2^) between SNPs were calculated by Haploview software, version 4.0 (Daly Lab at the Broad Institute, Cambridge, MA, USA).

To identify studies eligible for meta-analysis, MEDLINE citations (January 2000-January 2010) were surveyed using the National Library of Medicine's PubMed on-line search engine with each susceptibility gene name (*CDKAL1, IGF2BP2, SLC30A8, CDKN2A/B, HHEX, KCNQ1, FTO, TCF2*, and *WFS1*) and Chinese as key words. The retrieved references were read to identify studies that examined the allelic associations between the SNPs within the above nine genetic regions and T2D in Chinese Han living in China. For meta-analysis of *WFS1*, *FTO*, and *TCF2*, the C allele of *WFS1-*rs6446482, the A allele of *FTO-*rs8050136, and the T allele of *TCF2- *rs7501939 were used as surrogates for the minor A allele of *WFS1- *rs10010131, the A allele of *FTO-*rs9939609, and the G allele of *TCF2-*rs4430796 because they are in strong LD (r^2 ^= 1) in a HapMap Chinese Han population. In the meta-analysis, Cochran's x^2^-based Q-statistic test was performed to assess heterogeneity in all of the studies included, and a random-effect model was adopted when heterogeneity existed, otherwise a fixed-effects model was appropriate. Combined ORs were calculated using the Mantel-Haenszel (fixed-effects) and DerSimonian and Laird (random-effects) tests. The significant P value of overall ORs was determined using the Z-test. All calculations for the meta-analysis were performed on Revman42 (provided by Cochrane-Collaboration, available at http://ims.cochrane.org/revman/download/revman-4).

## Results

### Association study

We have successfully genotyped all nine SNPs. There were no significant departures from the Hardy-Weinberg equilibrium for all SNPs in the control group (p > 0.05) as assessed by a χ^2 ^test. In Table 2, we present for each SNP the genotype and allele distributions, risk allele-specific ORs, and p-values under an additive genetic model after correction for gender, age, and BMI. The allele frequencies of all SNPs in our studied population (Additional file 1, Table S1) are comparable to those of the Chinese Han population and different from Europeans in HapMap (http://www.hapmap.org/, accessed Feb 2009). The associations of 7 SNPs with T2D were observed, which included, as follows: *CDKAL1-*rs10946398 (OR, 1.27; 95% CI, 1.11-1.45; empirical p = 0.0008); *IGF2BP2-*rs4402960 (OR, 1.26; 95% CI, 1.08-1.47; empirical p = 0.003); *SLC30A8-*rs13266634(OR, 1.19; 95% CI,1.04-1.37; empirical p = 0.009); *CDKN2A/B-*rs10811661(OR, 1.22; 95% CI, 1.06-1.41; empirical p = 0.005); *HHEX-*rs5015480 (OR,1.20; 95% CI, 1.01-1.42; empirical p = 0.03); and *KCNQ1-*2237892 (OR, 1.37; 95% CI, 1.19-1.69; empirical p = 1.0 × 10^-4^). Interestingly, *FTO-*rs8050136 was also associated with T2D (OR, 1.25; 95% CI, 1.02-1.52; empirical p = 0.029), which remained significant after adjustment for age, gender, and BMI (OR,1.24; 95% CI, 1.01-1.52; empirical p = 0.046). However, no association with diabetes was found for *TCF2-*rs7501939 and *WFS1 *-rs6446482.

**Table 2:** Association with type 2 diabetes in the Chinese Han population.

Chr	SNPs	Genes	**Genotypes distribution **^**a**^		**Genotypic P**_**nominal**_	Minor/Major Alleles	RAF		Odd ratio (95%CI)	**Allelic P**_**nominal**_^**c**^	**Allelic P**_**permuted**_^**d**^
									
			Patients bb/Bb/BB	Controls Bb/Bb/BB			Patients	Controls			
9	rs10811661	*CDKN2A/B*	145:514:343	191:514:279	0.002	C/**T**	0.60	0.55	1.22(1.06-1.41)	0.005	0.005
8	rs13266634	*SLC30A8*	149:457:386	179:487:327	0.01	T/**C**	0.62	0.57	1.19(1.04-1.37)	0.009	0.009
4	rs6446482	*WFS1*	0:71:914	2:91:880	0.06	C/**G**	0.96	0.95	1.39(0.98-1.92)	0.06	0.06
10	rs5015480	*HHEX*	43:336:627	31:290:668	0.04	**C**/T	0.21	0.18	1.20(1.01-1.42)	0.03	0.03
17	rs7501939	*TCF2*	89:443:460	73:378:490	0.04	**T**/C	0.31	0.28	1.12(0.97-1.30)	0.12	0.12
6	rs10946398	*CDKAL1*	240:472:286	170:498:323	0.0006	**C**/A	0.48	0.42	1.27(1.11-1.45)	0.0006	0.0008
16	rs8050136	*FTO*	17:229:761	10:195:790	0.08	**A**/C	0.13	0.11	1.24(1.01-1.52)	0.041	0.046
3	rs4402960	*IGF2BP2*	76:418:503	53:356:571	0.001	**T**/G	0.29	0.24	1.26(1.08-1.47)	0.003	0.003
11	rs2237892	*KCNQ1*	69:396:525	107:437:415	1.23 × 10^-5^	T**/C**	0.73	0.66	1.37(1.19-1.69)	2.47 × 10^-5^	1.0 × 10^-4^

a. Genotype distributions are shown as the counts of three genotypes (bb, Bb, and BB). b, Minor allele; B, Major allele. RAF, Risk allele; OR, Risk allele-specific odds ratios calculated by logistic regression analysis. c. Adjusted for age, gender, and BMI. d. Permutation for 10,000 times. Bold indicates the risk allele.

### Meta-analysis

All published studies and references are shown in Additional file 1, Table S2, which indicates the inconsistency for associations between *HHEX, IGF2BP2, TCF2*, and *FTO *with T2D, the low MAF of *WFS1-*rs6446482 and *FTO-*rs8050136, and the small sample sizes in all studies in Chinese compared with those in Caucasian populations. Except for the Li study [13] due to the lack of allelic frequencies, the data from other studies and the current study were included in this meta-analysis to improve the power in evaluating the collective evidence on the relationships of these nine genetic loci to T2D, especially *HHEX, IGF2BP2, TCF2 FTO*, and *WFS1 *genetic loci. Table 3 shows the outcome of the meta-analysis. Overall, the pooled ORs for T2D were significant for *HHEX-*rs5015480 (pooled OR,1.24; *p *< 0.00001), *IGF2BP2-*rs4402960 (pooled OR, 1.14; *p *= 0.0002), *TCF2-*rs7501939 (pooled OR, 1.16; *p *< 0.00001), *FTO-*rs8050136 (pooled OR, 1.17; *p *< 0.00001), *WFS1-*rs6446482 (pooled OR, 1.26; *p *= 0.009), *CDKAL1-*rs10946398 (pooled OR, 1.24; *p *< 0.00001), *SLC30A8-*rs13266634 (pooled OR, 1.19; *p *< 0.00001), *CDKN2A/B-*rs10811661 (pooled OR, 1.28; *p *< 0.00001), and *KCNQ1-*2237892 (pooled OR, 1.33; *p *< 0.00001).

**Table 3:** Meta-analysis for SNPs at nine genetic loci in the Chinese Han population living in China.

Chr	SNP	Gene	Risk allele	Case risk AF	Control risk AF	*P *for heterogeneity	OR (95%CI)	*P *for overall effect	References
9	rs10811661	*CDKN2A/B*	T	0.60	0.54	0.07	1.28(1.21-1.36)	<0.00001	[10-12]
8	rs13266634	*SLC30A8*	C	0.58	0.52	0.10	1.19(1.13-1.25)	<0.00001	[6,10-12,27]
6	rs10946398	*CDKAL1*	C	0.45	0.41	0.03	1.24(1.13-1.37)	<0.00001	[10,12,15]
11	rs 2237892	*KCNQ1*	C	0.72	0.66	0.01	1.33(1.18-1.49)	<0.00001	[23-26]
4	rs6446482	*WFS1*	G	0.96	0.95	0.53	1.26(1.06-1.51)	0.009	[12]
10	rs5015480	*HHEX*	C	0.20	0.16	0.12	1.24(1.12-1.38)	<0.00001	[10,11]
17	rs7501939	*TCF2*	T	0.28	0.26	0.88	1.16(1.08-1.24)	<0.00001	[5,38]
3	rs4402960	*IGF2BP2*	T	0.27	0.24	0.22	1.14(1.06-1.22)	0.0002	[10,11,15]
16	rs8050136	*FTO*	A	0.14	0.12	0.80	1.17(1.09-1.27)	<0.00001	[11,12,14,30]

AF: allele frequency; OR: Odds ratio; CI: confidence interval; All of the calculations for the meta-analysis were carried out on Revman4.2. Cochran's x^2^-based Q-statistic test was used to assess heterogeneity in all studies, and a random-effect model was adopted when heterogeneity existed, otherwise a fixed-effects model was appropriate. Combined ORs were calculated using the Mantel-Haenszel (fixed-effects) and DerSimonian and Laird (random-effects) tests. The significant P value of the overall ORs was determined using a Z-test.

### Genotype-phenotype relationship analysis

*Genotype-age of diagnosis (AOD) for diabetes *For the patients with T2D, multiple linear regression was used to evaluate the effect of nine representative SNPs on AOD with adjustment for gender and BMI under *a priori *models. The analysis revealed that only *WFS1-*rs6446482 was negatively related to AOD under a dominant model (p = 0.02), and the patients with one or two risk alleles of *WFS1- *rs6446482 were diagnosed approximately 3 years earlier than those without risk alleles (49 ± 11 years versus 52 ± 11 years, p = 0.03). Prompted by this observation, we performed a subgroup analysis of 406 early-onset T2D patients (age of diagnosis ≦45 years) and 973 control subjects, showing that *WFS1- *rs6446482 was significantly associated with early-onset T2D (risk allele-specific OR, 1.96; 95% CI, 1.05-4.76; nominal p = 0.03; empiric p = 0.03) after adjustment for gender, age, and BMI.

*Genotype-BMI *For analysis of the genotype-BMI relationship, only the control subjects were examined because the treatment for T2D might distort the relationship. Only *FTO-*rs8050136 of nine genetic loci was found to be significantly associated with BMI (p = 0.04) after adjusting for gender and age as covariates. The individuals with one or two risk alleles had a higher BMI (25. ± 3.3 kg/m^2^, n = 205) than those without a risk allele (24.8 ± 3.4 kg/m^2^, n = 790; p = 0.04).

*Genotype-beta cell function and insulin resistance *Because the insulin level might be influenced by a hyperglycemic agent, 493 non-diabetic control subjects with fasting insulin concentrations available were studied. Only at the FTO and CDKAL1 loci was a relationship between SNP and phenotype observed. As shown in Table 4, the individuals with a risk allele of *FTO-*rs8050136 had a higher HOMA-ί, HOMA-IR, BMI, and fasting serum insulin level than those without the risk allele(under a dominant model), and the effects of *FTO *on HOMA-ί, HOMA-IR, and the fasting serum insulin level were independent of gender, age, and BMI. In addition, *CDKAL1-*rs10946398 significantly affected beta cell function independent of gender, age, and BMI, and the individuals with 2 risk alleles had a lower HOMA-ί than those without a risk allele (under the recessive model).

**Table 4:** The genotype-phenotype relationship in control individuals at CDKAL1 and FTO genetic loci.

SNPs	Genotype	Male/Female	Age (yrs)	**BMI (kg/m**^**2**^**)**	Fasting glucose (mmol/l)	Fasting insulin (pmol/l)	HOMA-ί	HOMA-IR
*CDKAL1 *rs10946398	AA	40/117	57 ± 8	25.1 ± 2.9	5.2 ± 0.4	50.1(34.1-71.7)	81.5(59.2-118.1)	1.6(1.2-2.5)
	AC	65/188	57 ± 8	25.1 ± 3.2	5.3 ± 0.4	46.7(36.9-66.9)	80.5(57.3-109.8)	1.5(1.1-2.3)
	CC	22/56	57 ± 9	25.9 ± 3.6	5.4 ± 0.4	48.1(36.9-64.8)	77.1(57.4-106.3)	1.6(1.2-2.3)
	Dominant p-value	-	-	0.650	0.140	0.268	0.142	0.268
	Additive p-value	-	-	0.131	0.161	0.525	**0.034(0.019)a**	0.155
	Recessive p-value	-	-	0.053	**0.044(0.046)a**	0.802	**0.043(0.021)a**	0.228
*FTO *rs8050136	CC	108/278	57 ± 8	25.1 ± 3.2	5.3 ± 0.4	45.3(32.7-63.4)	76.6(54.3-106.3)	1.5(1.1-2.2)
	CA/AA	19/85	57 ± 8	25.8 ± 3.2	5.2 ± 0.4	55.7(43.2-75.2)	94.3(73.5-129.6)	1.8(1.4-2.6)
	Dominant p value	-	-	**0.04(0.037)b**	0.148	**<0.00001(0.005)a**	**0.004(0.017)a**	**0.005(0.035)a**

Data are the means ± SD, or median(interquartile range). Test models refer to the minor allele. Only the dominant model was considered in which the minor allele homozygote count was ≤10. Bold: P-value < 0.05. Unadjusted p values without parentheses and p values with parentheses adjusted for a (gender, age, and BMI) and b (gender and age).

## Discussion

In the present study, we have replicated the associations between seven SNPs (*CDKAL1-*rs10946398, *IGF2BP2-*rs4402960, *SLC30A8-*rs13266634, *CDKN2A/B-*rs10811661, *HHEX-*rs5015480, *FTO-*rs8050136, and *KCNQ1-*rs2237892) with T2D in the Chinese Han population living in China, and an association between *WFS1-*rs6446482 with early-onset T2D was also identified in the subgroup analysis. For the genotype-phenotype analysis in control subjects, we found that the SNP at *FTO *loci showed a significant association with BMI, beta cell function, and insulin sensitivity. Moreover, we observed the influence of *CDKAL1 *on beta cell function, and our meta-analysis also indicated the SNPs at *TCF2-*rs7501939 and *WFS1-*rs6446482 were associated with T2D.

*SLC30A8, HHEX, CDKAL1, CDKN2A/B, IGF2BP2*, and *FTO *are among the first cohort of susceptibility genes discovered in relatively small samples by GWAS with ORs ranging from 1.14-1.20 [1-6]. In the present study, the OR values for the risk alleles range from 1.19-1.27, which are slightly higher than the Caucasian population [1-6], and slightly different from other Chinese studies. This is partly due to the features of our study (e.g., small sample sizes, enriched subjects with earlier onset T2D, and older subjects without a family history of T2D in the control group) or the difference in the studied population in genetic background as observed for the *TCF7L2 *and *KCNQ1 *genes in previous reports [7,21,22]. Based on our observation, we found that the minor allele frequencies of *FTO, WFS1, TCF2*, *HHEX*, and *IGF2BP2 *were much lower, while those of *CDKAL1, CDKN2A/B*, *SLC30A8*, and *KCNQ1 *were much higher than in the Caucasian population (Additional file 1, Table S1), indicating the different genetic background between Caucasians and the Chinese population. Our findings on *CDKAL1*, *SLC30A8*, *CDKN2A/B*, and *KCNQ1 *are further supported by several recent studies in the Chinese population [10-12,23-27]. However, for *IGF2BP2*, no significant association was demonstrated with T2D [10,11]. For *HHEX*, a relationship between the SNPs at the *HHEX *locus and T2D was reported among Chinese living in Shanghai, but not among Chinese in Beijing, even in the same study [10]. We believe that the inconsistent results of these studies are due, at least in part, to the small sample size [10] and the different enrollment criteria. For example, the plasma glucose at 2 hours during the OGTT was not determined and some subjects with impaired glucose tolerance(IGT) or T2D might have been falsely classified into the control group [10], or some adolescents in the control group may develop T2D in the future [11]. Therefore, the meta-analysis of the combined data from these published studies and our study is necessary for evaluating the association of these genetic loci with T2D and the effect of size in the Chinese population.

The studies in other ethnic groups have made clear that the association between *FTO *and T2D is mediated through its effect on body weight [2,3,28,29]. In the present study, we have confirmed the association between *FTO *and BMI and T2D, and the association with T2D remained of borderline significance (p = 0.046), even after adjustment for BMI. The independent effect of *FTO *on T2D is consistent with a recent study [14]. It is worth noticing that the study results on *FTO *in a Chinese population have been conflicting. The study in Chinese living in Taiwan has reported this association with BMI, but not T2D [30]. However, another study in a Chinese population living in Shanghai did not show an association between *FTO*-rs8050136 and BMI or T2D [13]. The discrepancy is partly due to population-specific bias, insufficient power, and the effects of duration and treatment for T2D, which may have distorted the real relationships among BMI, genotypes, and T2D. Europeans and Asians are different in their environmental risk profiles, body composition, and genetic background. In particular, Asians are at risk for T2D at a lower level of obesity, partly due to their increased predisposition to visceral adiposity [31] and reduced pancreatic β-cell function [32]. Our pooled analysis demonstrated *FTO *really contributed to T2D in Chinese. Unfortunately, the individual BMI from all published studies are not available for meta-analysis and so it is difficult to determine whether or not the association is really independent of BMI. As observed in the present study, *FTO-*rs8050136 may directly or indirectly affect HOMA-ί and HOMA-IR, and a reasonable interpretation is that the control subjects who carry the risk allele of *FTO *are prone to insulin resistance, and are able to increase insulin secretion to completely compensate for low insulin sensitivity to keep glucose homeostasis. Thus, it is necessary to clarify the pathogenesis of the contribution of *FTO *to T2D.

It has been shown that common SNPs at the WFS1 locus are clearly associated with T2D in a Caucasian population [8,33], but not in a Chinese population in a recent study with insufficient power [12]. However, we observed an association between *WFS1*-rs6446482 with T2D in our meta-analysis, and early-onset T2D in our own sample. Wolfram syndrome, characterized by early-onset diabetes, optic atrophy, deafness, and diabetes insipidus is due to mutations of the WFS1 gene [34], which regulates ί cell function in mice [35] and glucagon-like peptide 1-induced insulin secretion in humans [36], suggesting that impaired beta cell function in subjects with the risk allele of WFS1 may promote the onset of T2D as seen in other monogenetic diabetes [37,38]. Thus, it is necessary to replicate this study in a larger sample and sequence this gene for real causative variants.

The C allele of rs7501939 and the A allele of rs4430796 in the *TCF2 *gene were shown by GWAS to confer risk for prostate cancer and are protective for T2D [5]. However, that study did not show a significant association between *TCF2-*rs7501939 with T2D in a Chinese sample [5], consistent with the results of our study. The negative finding should be attributed to the insufficient power of this study (Additional file 1, Table S1). Nevertheless, in another Chinese study involving *TCF2-*rs4430796 [38], the association was replicated. A meta-analysis of combined data from both the previous study [38] and our study in Chinese confirmed the findings.

There are now as many as 17 genetic loci associated with T2D. In our previous Chinese Han studies [22,39,40], we have assessed the effects of *PPARG, KCNJ11*, and *TCF7L2*, and successfully replicated the associations between the *KCNJ11 *and *TCF7L2 *loci with T2D. However, the remaining six genetic loci were identified by meta-analysis of GWAS, including *JAZF1, TSPAN8-LGR5, THADA, ADAMTS9, NOTCH2-ADAM30, and MTNR1B*, of which only *MTNR1B *in one study was shown to increase the risk for T2D and an increased fasting plasma glucose concentration in the Chinese Han [12,41]. Therefore, further investigation with a large sample and meta-analysis is needed to confirm the roles in the Chinese Han population.

## Conclusions

In summary, the present study has confirmed an association between genetic variations at *CDKAL1, IGF2BP2, SLC30A8, CDKN2A/B, HHEX, KCNQ1*, and *FTO *loci with T2D in the Chinese Han population living in China. Our meta-analysis has provided the evidence that the variants at *TCF2 *and *WFS1 *genetic loci also contribute to the genetic susceptibility for T2D in Chinese.

## Competing interests

The authors declare that they have no competing interests.

## Authors' contributions

XYH carried out this study and drafted the manuscript. LNJ contributed to the design of the study and the revision of this manuscript. YYL, QR, XYZ, FW, XQS, and XHZ offered experimental, analytic, and data assistance. All authors have read and approved this final manuscript.

## Pre-publication history

The pre-publication history for this paper can be accessed here:

http://www.biomedcentral.com/1471-2350/11/81/prepub

## Supplementary Material

Additional file 1 Table S1 and Table S2. Click here for file

## References

[B1] Sladek R Rocheleau G Rung J Dina C Shen L Serre D Boutin P Vincent D Belisle A Hadjadj S Balkau B Heude B Charpentier G Hudson TJ Montpetit A Pshezhetsky AV Prentki M Posner BI Balding DJ Meyre D Polychronakos C Froguel P A genome-wide association study identifies novel risk loci for type 2 diabetes Nature 2007 445 881 885 10.1038/nature05616 17293876

[B2] Zeggini E Weedon MN Lindgren CM Frayling TM Elliott KS Lango H Timpson NJ Perry JR Rayner NW Freathy RM Barrett JC Shields B Morris AP Ellard S Groves CJ Harries LW Marchini JL Owen KR Knight B Cardon LR Walker M Hitman GA Morris AD Doney AS Wellcome Trust Case Control Consortium (WTCCC) Burton PR Clayton DG Craddock N Deloukas P Duncanson A Replication of genome-wide association signals in UK samples reveals risk loci for type 2 diabetes Science 2007 316 1336 1341 10.1126/science.1142364 17463249 PMC3772310

[B3] Scott LJ Mohlke KL Bonnycastle LL Willer CJ Li Y Duren WL Erdos MR Stringham HM Chines PS Jackson AU Prokunina-Olsson L Ding CJ Swift AJ Narisu N Hu T Pruim R Xiao R Li XY Conneely KN Riebow NL Sprau AG Tong M White PP Hetrick KN Barnhart MW Bark CW Goldstein JL Watkins L Xiang F Saramies J A genome-wide association study of type 2 diabetes in Finns detects multiple susceptibility variants Science 2007 316 1341 1345 10.1126/science.1142382 17463248 PMC3214617

[B4] Saxena R Voight BF Lyssenko V Burtt NP de Bakker PI Chen H Roix JJ Kathiresan S Hirschhorn JN Daly MJ Hughes TE Groop L Altshuler D Almgren P Florez JC Meyer J Ardlie K Bengtsson Bostrom K Isomaa B Lettre G Lindblad U Lyon HN Melander O Newton-Cheh C Nilsson P Orho-Melander M Rastam L Speliotes EK Taskinen MR Tuomi T Genome-wide association analysis identifies loci for type 2 diabetes and triglyceride levels Science 2007 316 1331 1336 10.1126/science.1142358 17463246

[B5] Gudmundsson J Sulem P Steinthorsdottir V Bergthorsson JT Thorleifsson G Manolescu A Rafnar T Gudbjartsson D Agnarsson BA Baker A Sigurdsson A Benediktsdottir KR Jakobsdottir M Blondal T Stacey SN Helgason A Gunnarsdottir S Olafsdottir A Kristinsson KT Birgisdottir B Ghosh S Thorlacius S Magnusdottir D Stefansdottir G Kristjansson K Bagger Y Wilensky RL Reilly MP Morris AD Kimber CH Two variants on chromosome 17 confer prostate cancer risk, and the one in TCF2 protects against type 2 diabetes Nat Genet 2007 39 77 983 10.1038/ng2062 17603485

[B6] Steinthorsdottir V Thorleifsson G Reynisdottir I Benediktsson R Jonsdottir T Walters GB Styrkarsdottir U Gretarsdottir S Emilsson V Ghosh S Baker A Snorradottir S Bjarnason H Ng MC Hansen T Bagger Y Wilensky RL Reilly MP Adeyemo A Chen Y Zhou J Gudnason V Chen G Huang H Lashley K Doumatey A So WY Ma RC Andersen G Borch-Johnsen K A variant in CDKAL1 influences insulin response and risk of type 2 diabetes Nat Genet 2007 39 770 775 10.1038/ng2043 17460697

[B7] Yasuda K Miyake K Horikawa Y Hara K Osawa H Furuta H Hirota Y Mori H Jonsson A Sato Y Yamagata K Hinokio Y Wang HY Tanahashi T Nakamura N Oka Y Iwasaki N Iwamoto Y Yamada Y Seino Y Maegawa H Kashiwagi A Takeda J Maeda E Shin HD Cho YM Park KS Lee HK Ng MC Ma RC Variants in KCNQ1 are associated with susceptibility to type 2 diabetes mellitus Nat Genet 2008 40 1092 1097 10.1038/ng.207 18711367

[B8] Sandhu MS Weedon MN Fawcett KA Wasson J Debenham SL Daly A Lango H Frayling TM Neumann RJ Sherva R Blech I Pharoah PD Palmer CN Kimber C Tavendale R Morris AD McCarthy MI Walker M Hitman G Glaser B Permutt MA Hattersley AT Wareham NJ Barroso I Common variants in WFS1 confer risk of type 2 diabetes Nat Genet 2007 39 951 953 10.1038/ng2067 17603484 PMC2672152

[B9] Zeggini E Scott LJ Saxena R Voight BF Marchini JL Hu T de Bakker PI Abecasis GR Almgren P Andersen G Ardlie K Boström KB Bergman RN Bonnycastle LL Borch-Johnsen K Burtt NP Chen H Chines PS Daly MJ Deodhar P Ding CJ Doney AS Duren WL Elliott KS Erdos MR Frayling TM Freathy RM Gianniny L Grallert H Grarup N Meta-analysis of genome-wide association data and large-scale replication identifies additional susceptibility loci for type 2 diabetes Nat Genet 2008 40 638 645 10.1038/ng.120 18372903 PMC2672416

[B10] Wu Ying Huaixing Li Loos Ruth JF Yu Zhijie Ye Xingwang Chen Lihua Pan An Hu Frank B Lin Xu Common variants in CDKAL1, CDKN2A/B, IGF2BP2, SLC30A8, and HHEX/IDE genes are associated with type 2 diabetes and impaired fasting glucose in a Chinese Han population Diabetes 2008 57 2834 2842 10.2337/db08-0047 18633108 PMC2551696

[B11] Ng Maggie CY Park Kyong Soo Oh Bermseok Tam Claudia HT Cho Young Min Shin Hyoung Doo Lam Vincent KL Ma Ronald CW So Wing Yee Cho Yoon Shin Kim Hyung-Lae Lee Hong Kyu Chan Juliana CN Cho Nam H Implications of genetic variants near TCF7L2, SLC30A8, HHEX, CDKAL1, CDKN2A/B, IGF2BP2, and FTO in type 2 diabetes and obesity in 6,719 Asians Diabetes 2008 57 2226 2233 10.2337/db07-1583 18469204 PMC2494677

[B12] Hu C Zhang R Wang C Wang J Ma X Lu J Qin W Hou X Wang C Bao Y Xiang K Jia W PPARG, KCNJ11, CDKAL1, CDKN2A-CDKN2B, IDE-KIF11-HHEX, IGF2BP2 and SLC30A8 are associated with type 2 diabetes in a Chinese population PLoS One 2009 4 e7643 10.1371/journal.pone.0007643 19862325 PMC2763267

[B13] Li H Wu Y Loos RJ Hu FB Liu Y Wang J Yu Z Lin X Variants in the fat mass- and obesity-associated (FTO) gene are not associated with obesity in a Chinese Han population Diabetes 2008 57 264 268 10.2337/db07-1130 17959933

[B14] Liu Y Liu Z Song Y Zhou D Zhang D Zhao T Chen Z Yu L Yang Y Feng G Li J Zhang J Liu S Zhang Z He L Xu H Meta-analysis Added Power to Identify Variants in FTO Associated With Type 2 Diabetes and Obesity in the Asian Population Obesity (Silver Spring) 2010 advance online publication 10.1038/oby.2009.469 20057365

[B15] Liu Y Yu L Zhang D Chen Z Zhou DZ Zhao T Li S Wang T Hu X Feng GY Zhang ZF He L Xu H Positive association between variations in CDKAL1 and type 2 diabetes in Han Chinese individuals Diabetologia 2008 51 2134 2137 10.1007/s00125-008-1141-6 18766326

[B16] Alberti KG Zimmet PZ Definition, diagnosis and classification of diabetes mellitus and its complications. Part 1: diagnosis and classification of diabetes mellitus provisional report of a WHO consultation Diabet Med 1998 15 539 553 10.1002/(SICI)1096-9136(199807)15:7<539::AID-DIA668>3.0.CO;2-S 9686693

[B17] Han XY Ji LN Contribution of MODY2 gene to the pathogenesis of Chinese early onset familial type 2 diabetes Beijing Da Xue Xue Bao 2005 18 591 594 16378108

[B18] Han XY Liu CY Ji LN Contribution of MODY6 gene in the pathogenesis of familial type 2 diabetes in Chinese population Zhonghua Yi Xue Za Zhi 2005 14 2463 2467 16321269

[B19] Matthews RD Hosker JP Rudenski AS Naylor BA Treacher DF Turner RC Homeostasis model assessment: insulin resistance and beta cell function from fasting plasma glucose and insulin concentrations in man Diabetologia 1985 28 412 419 10.1007/BF00280883 3899825

[B20] Gu D Reynolds K Duan X Xin X Chen J Wu X Mo J Whelton PK He J InterASIA Collaborative Group Prevalence of diabetes and impaired fasting glucose in the Chinese adult population: International Collaborative Study of Cardiovascular Disease in Asia (InterASIA) Diabetologia 2003 46 1190 1198 10.1007/s00125-003-1167-8 12879248

[B21] Ng MC Tam CH Lam VK So WY Ma RC Chan JC Replication and identification of novel variants at TCF7L2 associated with type 2 diabetes in Hong Kong Chinese J Clin Endocrinol Metab 2007 92 3733 3737 10.1210/jc.2007-0849 17609304

[B22] Ren Q Han XY Wang F Zhang XY Han LC Luo YY Zhou XH Ji LN Exon sequencing and association analysis of polymorphisms in TCF7L2 with type 2 diabetes in a Chinese population Diabetologia 2008 51 1146 1152 10.1007/s00125-008-1039-3 18493736

[B23] Hu C Wang C Zhang R Ma X Wang J Lu J Qin W Bao Y Xiang K Jia W Variations in KCNQ1 are associated with type 2 diabetes and beta cell function in a Chinese population Diabetologia 2009 52 1322 1325 10.1007/s00125-009-1335-6 19308350

[B24] Liu Y Zhou DZ Zhang D Chen Z Zhao T Zhang Z Ning M Hu X Yang YF Zhang ZF Yu L He L Xu H Variants in KCNQ1 are associated with susceptibility to type 2 diabetes in the population of mainland China Diabetologia 2009 52 1315 1321 10.1007/s00125-009-1375-y 19448982 PMC2688614

[B25] Qi Q Li H Loos RJ Liu C Wu Y Hu FB Wu H Lu L Yu Z Lin X Common variants in KCNQ1 are associated with type 2 diabetes and impaired fasting glucose in a Chinese Han population Hum Mol Genet 2009 18 3508 3515 10.1093/hmg/ddp294 19556355

[B26] Chen Z Zhang X Ma G Qian Q Yao Y Association study of four variants in KCNQ1 with type 2 diabetes mellitus and premature coronary artery disease in a Chinese population Mol Biol Rep 2010 37 207 212 10.1007/s11033-009-9597-0 19575309

[B27] Xiang J Li XY Xu M Hong J Huang Y Tan JR Lu X Dai M Yu B Ning G Zinc transporter-8 gene (SLC30A8) is associated with type 2 diabetes in Chinese J Clin Endocrinol Metab 2008 93 4107 4112 10.1210/jc.2008-0161 18628523

[B28] Frayling TM Timpson NJ Weedon MN Zeggini E Freathy RM Lindgren CM Perry JR Elliott KS Lango H Rayner NW Shields B Harries LW Barrett JC Ellard S Groves CJ Knight B Patch AM Ness AR Ebrahim S Lawlor DA Ring SM Ben-Shlomo Y Jarvelin MR Sovio U Bennett AJ Melzer D Ferrucci L Loos RJ Barroso I Wareham NJ A common variant in the FTO gene is associated with body mass index and predisposes to childhood and adult obesity Science 2007 316 889 894 10.1126/science.1141634 17434869 PMC2646098

[B29] Scuteri A Sanna S Chen WM Uda M Albai G Strait J Najjar S Nagaraja R Orr M Usala G Dei M Lai S Maschio A Busonero F Mulas A Ehret GB Fink AA Weder AB Cooper RS Galan P Chakravarti A Schlessinger D Cao A Lakatta E Abecasis GR Genome-wide association scan shows genetic variants in the FTO gene are associated with obesity-related traits PLoS Genet 2007 3 1200 1210 10.1371/journal.pgen.0030115 PMC1934391 17658951

[B30] Chang YC Liu PH Lee WJ Chang TJ Jiang YD Li HY Kuo SS Lee KC Chuang LM Common variation in the fat mass and obesity-associated (FTO) gene confers risk of obesity and modulates BMI in the Chinese population Diabetes 2008 57 2245 2252 10.2337/db08-0377 18487448 PMC2494679

[B31] Yoon KH Lee JH Kim JW Cho JH Choi YH Ko SH Zimmet P Son HY Epidemic obesity and type 2 diabetes in Asia Lancet 2006 368 1681 1688 10.1016/S0140-6736(06)69703-1 17098087

[B32] Torrens JI Skurnick J Davidow AL Korenman SG Santoro N Soto-Greene M Lasser N Weiss G Ethnic differences in insulin sensitivity and β-cell function in premenopausal or early perimenopausal women without diabetes: the Study of Women's Health Across the Nation (SWAN) Diabetes Care 2004 27 354 361 10.2337/diacare.27.2.354 14747213

[B33] Franks PW Rolandsson O Debenham SL Fawcett KA Payne F Dina C Froguel P Mohlke KL Willer C Olsson T Wareham NJ Hallmans G Barroso I Sandhu MS Replication of the association between variants in WFS1 and risk of type 2diabetes in European populations Diabetologia 2008 51 458 463 10.1007/s00125-007-0887-6 18040659 PMC2670195

[B34] Ishihara H Takeda S Tamura A Takahashi R Yamaguchi S Takei D Yamada T Inoue H Soga H Katagiri H Tanizawa Y Oka Y Disruption of the WFS1 gene in mice causes progressive beta-cell loss and impaired stimulus-secretion coupling in insulin secretion Hum Mol Genet 2004 13 1159 1170 10.1093/hmg/ddh125 15056606

[B35] Inoue H Tanizawa Y Wasson J Behn P Kalidas K Bernal-Mizrachi E Mueckler M Marshall H Donis-Keller H Crock P Rogers D Mikuni M Kumashiro H Higashi K Sobue G Oka Y Permutt MA A gene encoding a transmembrane protein is mutated in patients with diabetes mellitus and optic atrophy (Wolfram syndrome) Nat Genet 1998 20 143 148 10.1038/2441 9771706

[B36] Schäfer SA Müssig K Staiger H Machicao F Stefan N Gallwitz B Häring HU Fritsche A A common genetic variant in WFS1 determines impaired glucagon-like peptide 1 induced insulin secretion Diabetologia 2009 52 1075 1082 10.1007/s00125-009-1344-5 19330314

[B37] Lehman DM Richardson DK Jenkinson CP Hunt KJ Dyer TD Leach RJ Arya R Abboud HE Blangero J Duggirala R Stern MP P2 promoter variants of the hepatocyte nuclear factor 4alpha gene are associated with type 2 diabetes in Mexican Americans Diabetes 2007 56 513 517 10.2337/db06-0881 17259399

[B38] Wang C Hu C Zhang R Bao Y Ma X Lu J Qin W Shao X Lu J Xu J Lu H Xiang K Jia W Common variants of hepatocyte nuclear factor 1beta are associated with type 2 diabetes in a Chinese population Diabetes 2009 58 1023 1027 10.2337/db08-1064 19168595 PMC2661584

[B39] Wang F Han XY Ren Q Zhang XY Han LC Luo YY Zhou XH Ji LN Effect of genetic variants in KCNJ11, ABCC8, PPARG and HNF4A loci on the susceptibility of type 2 diabetes in Chinese Han population Chin Med J (Engl) 2009 122 2477 2482 20079163

[B40] Luo Y Wang H Han X Ren Q Wang F Zhang X Sun X Zhou X Ji L Meta-analysis of the association between SNPs in TCF7L2 and type 2 diabetes in East Asian population Diabetes Res Clin Pract 2009 85 139 146 10.1016/j.diabres.2009.04.024 19482368

[B41] Rönn T Wen J Yang Z Lu B Du Y Groop L Hu R Ling C A common variant in MTNR1B, encoding melatonin receptor 1B, is associated with type 2 diabetes and fasting plasma glucose in Han Chinese individuals Diabetologia 2009 52 830 833 10.1007/s00125-009-1297-8 19241057

