# Supplementary material for: Implication of genetic variants near SLC30A8, HHEX, CDKAL1, CDKN2A/B, IGF2BP2, FTO, TCF2, KCNQ1, and WFS1 in Type 2 Diabetes in a Chinese population
Source: BMC Med Genet. 2010 May 28;11:81. doi: 10.1186/1471-2350-11-81 (PMC2896346; doi:10.1186/1471-2350-11-81)
Supplement: Additional file 1 — Table S1 and Table S2. [file 1471-2350-11-81-S1.DOC]

**Table S1 The power and minor allele frequencies in the current study and different ethnic groups from HapMap**

| SNP ID | genes | Minor/  Major allele | ORs previously reported(Refs) | Powers | Minor allele frequencies (HapMap) | | | | |
| --- | --- | --- | --- | --- | --- | --- | --- | --- | --- |
|  |  |  |  |  | This CHB JPT CEU *P*  Sample | | | | |
| rs10811661 | *CDKN2A/B* | C/**T** | 1.20(2) | 0.82 | 0.45 | 0.42 | 0.44 | 0.20 | <0.00001 |
| rs13266634 | *SLC30A8* | T/**C** | 1.18(1) | 0.73 | 0.43 | 0.47 | 0.44 | 0.24 | <0.00001 |
| rs6446482 | *WFS1* | C/**G** | 1.15(8) | 0.15 | 0.05 | 0.04 | 0.01 | 0.26 | <0.00001 |
| rs5015480 | *HHEX* | **C**/T | 1.13(2) | 0.33 | 0.18 | 0.21 | 0.19 | 0.45 | 0.0002 |
| rs7501939 | *TCF2* | **T**/C | 1.19(5) | 0.70 | 0.28 | 0.23 | 0.29 | 0.43 | <0.00001 |
| rs10946398 | *CDKAL1* | **C**/A | 1.12(2) | 0.43 | 0.42 | 0.42 | 0.41 | 0.34 | 0.09 |
| rs8050136 | *FTO* | **A**/C | 1.11(3) | 0.18 | 0.11 | 0.14 | 0.18 | 0.46 | <0.00001 |
| rs4402960 | *IGF2BP2* | **T**/G | 1.14(2) | 0.43 | 0.24 | 0.24 | 0.30 | 0.30 | <0.00001 |
| rs2237892 | *KCNQ1* | T**/C** | 1.40(7) | 0.95 | 0.34 | 0.35 | 0.36 | 0.08 | <0.00001 |

Power calculations were performed using Quanto software (available at http://hydra.usc.edu/gxe/) using the odds ratios from published studies, sample sizes , minor allele frequencies in the present study, and the prevalence of type 2 diabetes in China (20). The alleles in bold are the risk alleles for type 2 diabetes identified in the present study. *p* represents the significance of the differences between the CEU and CHB populations analyzed by Pearson’s χ^2^ test..

**Table S2. The published association studies of nine genetic loci in** Chinese Han population living in China

| Gene | SNPs | References | Number  (case/control) | RA | RAF  case | RAF  control | OR(95%CI) | *P* values |
| --- | --- | --- | --- | --- | --- | --- | --- | --- |
| *CDKAL1* | rs10946398 | 10 | 424/1908 | C | 0.50 | 0.41 | 1.47(1.25-1.73) | 2.3×10^-6^ |
|  |  | 12 | 1849/1785 | C | 0.44 | 0.41 | 1.114(1.014-1.224) | 0.0074 |
|  |  | 15 | 1822/1903 | C | 0.44 | 0.39 | 1.25(1.13-1.38) | 8.27 x 10^–6^ |
| *CDKN2A/B* | rs10811661 | 10 | 424/1908 | T | 0.58 | 0.52 | 1.31(1.12-1.54) | 0.001 |
|  |  | 11 | 1481/1530 | T | 0.61 | 0.57 | 1.21 (1.09–1.34) | 3.5×10^-4^ |
|  |  | 12 | 1849/1785 | T | 0.60 | 0.52 | 1.406(1.280-1.546) | 1.13×10^-13^ |
| *SLC30A8* | rs13266634 | 6 | 1426/970 | C | 0.57 | 0.52 | 1.19(1.06-1.33) | 0.0035 |
|  |  | 10 | 424/1908 | C | 0.58 | 0.57 | 1.09(0.93-1.27) | 0.28 |
|  |  | 11 | 1481/1530 | C | 0.57 | 0.53 | 1.17（1.06-1.3） | 0.02 |
|  |  | 12 | 1849/1785 | C | 0.61 | 0.56 | 1.251(1.138-1.374) | 1.60×10^-6^ |
|  |  | 27 | 721/521 | C | 0.58 | 0.53 | 1.22 (1.04–1.43) | 0.016 |
| *IGF2BP2* | rs4402960 | 10 | 424/1908 | T | 0.26 | 0.24 | 1.14(0.95-1.35) | 0.16 |
|  |  | 11 | 1481/1530 | T | 0.26 | 0.25 | 1.05（0.93-1.18） | 0.41 |
|  |  | 15 | 1863/1970 | T | 0.26 | 0.25 | 1.06(0.95-1.18) | 0.29 |
| *HHEX* | rs5015480 | 10 | 272/723 | C | 0.20 | 0.19 | 1.13(0.88-1.46) | 0.33 |
|  |  | 10 | 152/1185 | C | 0.22 | 0.14 | 1.79(1.30-1.94) | 0.0003 |
|  |  | 11 | 1481/1530 | C | 0.19 | 0.17 | 1.09(0.95-1.25) | 0.228 |
| *TCF2* | rs7501939 | 5 | 1495/993 | T | 0.23 | 0.21 | 1.15 (1.00–1.32) | 0.054 |
|  | rs 4430796 | 38 | 1859/1785 | G | 0.31 | 0.28 | 1.13(1.02-1.26) | 0.0178 |
| *FTO* | rs8050136 | 11 | 1481/1530 | A | 0.16 | 0.14 | 1.18 (1.02–1.37 | 0.028 |
|  |  | 12 | 1849/1785 | A | 0.13 | 0.12 | 1.13(0.98-1.29) | 0.1456 |
|  |  | 13 | 424/1908 | A | not shown | not shown | 0.91(0.71-1.16) | 0.43 |
|  |  | 14 | 1748/2015 | A | 0.14 | 0.12 | 1.22(1.05-1.41) | 0.008 |
|  | rs9939609 | 30 | 238/726 | A | 0.13 | 0.13 | 1.05(0.84-1.31) | 1.0 |
| *WFS1* | rs10010131 | 12 | 1849/1785 | A | 0.96 | 0.95 | 1.21(0.98-1.51) | 0.0969 |
| *KCNQ1* | rs2237892 | 23 | 1719/1720 | C | 0.74 | 0.65 | 1.53(1.38-1.70) | 2.4×10^-16^ |
|  |  | 24 | 1880/1996 | C | 0.70 | 0.67 | 1.23(1.11-1.36) | 1.13.5×10^-4^ |
|  |  | 25 | 424/1908 | C | 0.73 | 0.68 | 1.36(1.15-1.62) | 0.0004 |
|  |  | 26 | 57/341 | C | 0.69 | 0.68 | 1.15(0.41-3.27) | 0.788 |

OR: Risk allele specific odds ratio after adjustment for age, gender, and BMI except for reference 11; RA: Risk allele; RAF: Risk allele frequency
